# Electrochemotherapy and Other Clinical Applications of Electroporation for the Targeted Therapy of Metastatic Melanoma

**DOI:** 10.3390/ma14143985

**Published:** 2021-07-16

**Authors:** Corina Ioana Cucu, Călin Giurcăneanu, Liliana Gabriela Popa, Olguța Anca Orzan, Cristina Beiu, Alina Maria Holban, Alexandru Mihai Grumezescu, Bogdan Mircea Matei, Marius Nicolae Popescu, Constantin Căruntu, Mara Mădălina Mihai

**Affiliations:** 1 Department of Oncologic Dermatology-“Elias” Emergency University Hospital, “Carol Davila” University of Medicine and Pharmacy, 020021 Bucharest, Romania; corina-ioana.cucu@drd.umfcd.ro (C.I.C.); calin.giurcaneanu@umfcd.ro (C.G.); olguta.orzan@umfcd.ro (O.A.O.); cristina.popescu@drd.umfcd.ro (C.B.); mara.mihai@umfcd.ro (M.M.M.); 2 Department of Microbiology and Immunology, Faculty of Biology, University of Bucharest, 030018 Bucharest, Romania; alina.m.holban@bio.unibuc.ro; 3 Research Institute of the University of Bucharest, 050657 Bucharest, Romania; 4 Department of Science and Engineering of Oxide Materials and Nanomaterials, Faculty of Applied Chemistry and Materials Science, University Politehnica of Bucharest, 1-7 Polizu Street, 011061 Bucharest, Romania; grumezescu@yahoo.com; 5 Department of Biophysics and Cellular Biotechnology, “Carol Davila” University of Medicine and Pharmacy, 020021 Bucharest, Romania; bogdan.matei@umfcd.ro; 6 Department of Physical and Rehabilitation Medicine, “Elias” Emergency University Hospital, “Carol Davila” University of Medicine and Pharmacy, 020021 Bucharest, Romania; marius_drm1987@yahoo.com; 7 Faculty of Medicine, “Titu Maiorescu” University, 22 Dambrovnicului, 031593 Bucharest, Romania; costin.caruntu@gmail.com

**Keywords:** electrochemotherapy, electropermeabilization, electroporation, metastatic melanoma, advanced melanoma, targeted therapy, gene transfer

## Abstract

Electrochemotherapy (ECT) is an effective bioelectrochemical procedure that uses controlled electrical pulses to facilitate the increase of intracellular concentration of certain substances (electropermeabilization/ reversible electroporation). ECT using antitumor drugs such as bleomycin and cisplatin is a minimally invasive targeted therapy that can be used as an alternative for oncologic patients not eligible for surgery or other standard therapies. Even though ECT is mainly applied as palliative care for metastases, it may also be used for primary tumors that are unresectable due to size and location. Skin neoplasms are the main clinical indication of ECT, the procedure reporting good curative results and high efficiency across all tumor types, including melanoma. In daily practice, there are many cases in which the patient’s quality of life can be significantly improved by a safe procedure such as ECT. Its popularity must be increased because it has a safe profile and minor local adverse reactions. The method can be used by dermatologists, oncologists, and surgeons. The aim of this paper is to review recent literature concerning electrochemotherapy and other clinical applications of electroporation for the targeted therapy of metastatic melanoma.

## 1. Introduction

The main cause of death in oncology is represented by cancer progression through metastasis, with the dissemination to secondary organs [1,2], and significant efforts are being made to the development of novel therapeutic strategies to combat metastatic cancer [3].

Electrochemotherapy (ECT) is an effective bioelectrochemical procedure that uses controlled electrical pulses (electropermeabilization) to facilitate the increase of intracellular concentration of certain substances. It combines low-dose chemotherapy with reversible electroporation [4].

The history of electrochemotherapy (ECT) began in 1957 when the impact of electric fields on cell membranes was discovered [5]. When a biological surface is exposed to an exceedingly high external electric field, the surface’s permeability, and conductivity rise immediately. The purpose of reversible electroporation is to transiently modify the permeability of the cell membrane and its surface tension by dints of short, high-voltage electric pulses. While the exact process is not completely understood, the exposure of the cellular membrane to an electric field destabilizes the phospholipid bilayer, thus creating aqueous pores [6,7,8,9,10,11,12]. Chemotherapeutic drugs are administered either intratumorally or intravenous, reaching lower systemic concentrations than those of standard oncologic regimens, and, therefore, they have reduced drug-related side effects. Electropermeabilization increases the intracellular uptake of chemotherapeutic drugs and enhances their tumoral cytotoxicity [13,14].

Solid tumor cells develop various mechanisms to block intracellular drug delivery. Reversible electroporation facilitates the chemical substance to penetrate cellular membranes, which is useful in the case of chemotherapeutic agents with low permeability. After these molecules achieve optimal intracellular concentrations, they exert their effect on the targeted tumor cells. The process is reversible since the cell membrane becomes stable afterward, without affecting cell viability.

Bleomycin (BLM) and cisplatin (CDDP) have a hydrophilic structure, with poor ability to cross cell membranes [15]. These two drugs have shown the best results when administered intravenously or intratumorally at appropriate time moments prior to applying local electric pulses to be present in an efficient concentration outside the cell when its membrane is permeabilized by the electric field [16,17]. Multiple types of electrodes exist, but standard ECT fixed-geometry electrodes are mainly used in cutaneous malignancies.

The use of ECT is based on comprehensive preclinical and clinical studies. This strategy of treating specific tumors is gaining popularity around Europe, where more than 150 ECT centers exist, and large studies were conducted [18,19]. The European Standard Operating Procedures of ECT (ESOPE) project established the guidelines for a safe application of ECT in clinical practice that began with its use in 2006 [20].

Even though it is mainly applied as palliative care for metastases, it may also be used for primary tumors that are unresectable due to size and location. Skin neoplasms are the main clinical indication of ECT, the procedure reporting good curative results and high efficiency across all tumor types, including melanoma. Scientific reports estimate that 2–18% of patients diagnosed with melanoma develop cutaneous or subcutaneous metastasis [4,21,22], while 42–60% of total cases for metastatic melanoma are defined by skin metastatic dissemination [4,21,22,23,24,25]. Managing the patients is difficult at this stage, with limited options, and requires a complex multidisciplinary approach.

The aim of this paper is to review recent literature concerning electrochemotherapy use in metastatic melanoma.

## 2. Mechanism of Action of Electrochemotherapy

The therapeutic agent can be administered intralesional or intravenous, reaching lower systemic concentrations compared with standard chemotherapeutic regimens [14].

The electric pulses applied to the tissue cause an arteriolar vasoconstriction reflex that ends after two minutes. Consequently, temporary local hypoperfusion and interstitial edema occur, processes that are reversible after the cell membrane reseals. The effect is more intense in karyokinetic cells of an immature endothelium, which can last up to four days, especially when the interstitial pressure is high [26]. The vascular changes also named the “vascular lock”, are mediated by the sympathetic nervous system and are affected by the duration of drug administration [27]. The procedure consists of retaining the drug in the tumor cells while the entry of the cytostatic agent in the bloodstream is impaired [28]. This effect is more durable in neoplastic tissues by decreasing blood washout, thus increasing tumor susceptibility to chemotherapy.

A group of ion pumps and channels causes a potential physiological difference along every cell plasma membrane. When an external electric field is applied to the cell, a supplementary potential is added to the cell membrane, leading to a distinct transmembrane potential difference since the electric field is present [29]. The induced transmembrane voltage is directly proportional to the level of the external electric field; subsequently, hydrophilic (aqueous) pores are formed [30]. While the amplitude of the electric field increases, the aqueous pores become more stable because of the disconnection of the lipids in the membrane bilayer, leading to the development of nanosized pores [31]. However, the pores are transient and disappear within a few seconds to several minutes by membrane resealing [32].

**Figure 1:**
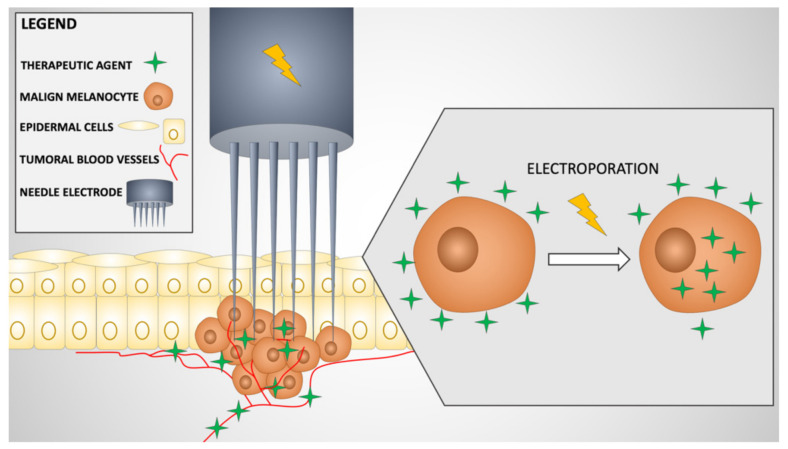
Flow chart presenting steps in electrochemotherapy in melanoma. Initially, the therapeutic agent is administered intravenously or intratumoral. The drug progressively surrounds the tumoral cells and achieves optimal local concentrations. The needle electrode is inserted into the targeted tissue (in particular, melanoma metastasis), and the generator applies electric pulses leading to a “vascular lock”, with temporary local hypoperfusion and interstitial edema; increased permeability of cell membranes in malignant melanocytes, with an enhanced intracellular uptake of the drug and cytotoxicity.

The term electroporation was chosen for this phenomenon because, after the cells are treated with electric pulses, particles that usually do not pass through the membrane diffuse to the cytosol (Figure 1) [33]. While the carefully modulated electric field transient increases cell permeability, the toxicity of a cytostatic agent is multiplied by the fact that sufficient quantities of the drug infiltrate the intracellular compartments of tumor cells [34]. The cytotoxicity of these drugs increases from two to several thousand folds [35].

After membrane resealing, cytostatic drugs selectively eliminate tumoral cells with faster division rates than normal cells surrounding the tumor [36]. Depending on the doses of the drug (BLM in particular), two mechanisms of tumor cell death have been described: Low doses were associated with apoptosis by the appearance of atypical mitoses, whereas higher doses trigger a pseudo-apoptotic pathway since the drug induces alterations of double-strand DNA [37]. ECT also leads to immunogenic cell death since the released tumor-associated antigens can trigger an immune response. This effect may also be enhanced by the addition of immunotherapy to ECT [4].

In summary, three main biological situations are converging toward an antitumor effect of ECT. The first one directly increased cytotoxicity determined by the electric pulse-controlled delivery of the drug to the tumor cells [3]. A plentiful amount of drug should be delivered to the tumor cells, with total coverage of the cutaneous lesions, in order for ECT to be efficacious [38]. The second deals with the vasoconstrictive effect of the precapillary sphincters due to electric pulses that block the cytotoxic in the tumor. Moreover, it has been reported that ECT has the capacity to remove tumor vascularization, also called “vascular disrupting action” [39]. Finally, ECT stimulates the immune response. Both BLM and CDDP produce immunologic cell death by releasing damage-associated molecules [40], stimulating the local antitumoral immunity [41]; this leads to the assumption that ECT can transform the tumor into an “in situ” vaccine, but further research must be continued [42].

## 3. Electrochemotherapy Treatment Regimen

The ECT procedure inclusively for skin melanoma metastases is detailed in the published studies of ESOPE (European Standard Operating Procedures of Electrochemotherapy) and SOP (Standard Operating Procedures), the treatment being performed according to these standards [20,26,43]. The online platform International Network for Sharing Practices on Electrochemotherapy (InspECT) also offers standardized information on the use of ECT [44], while the National Institute for Health and Care Excellence (NICE) has issued guidance for the United Kingdom [45]. Different combinations of cytostatic drugs were studied in electroporation, both in preclinical and clinical trials. These studies considered several chemotherapeutic drugs, such as bleomycin, cisplatin, carboplatin, mitomycin-C, actinomycin D, adriamycin, cyclophosphamide, daunorubicin, doxorubicin, etoposide, paclitaxel, 5-fluorouracil, vinblastine, vincristine, gemcitabine, netropsin, cytarabine, oxaliplatin, methotrexate, melphalan, ancitabine, taxotere, and nimustine [26,46]. Nevertheless, the highest rate of cytotoxicity was observed for bleomycin, which expanded toxicity by up to 1000 times; for cisplatin, up to 80 times in studies in vitro [34].

Bleomycin is the drug most frequently used in association with electroporation, but ECT standing on cisplatin is equivalently effective. Few clinical studies are available outlining the use of cisplatin, primarily due to the requirement of administering this cytostatic agent directly into the tumor [47]. This kind of administration is not feasible when a patient has disseminated skin lesions [16]. If the patient has small, single lesions (diameter smaller than 2 cm of each lesion and less than seven lesions), both bleomycin and cisplatin can be administered locally, intratumorally [20,43].

By considering the extent of the procedure, both local and general anesthesia may be performed. The chemotherapeutic agent is administered at the beginning, and after waiting one minute, electroporation is performed. If the patient’s skin lesions are above 2 cm in diameter or there are more than seven lesions, bleomycin administered intravenously is the most efficient cytostatic drug [20,43]. As before, the procedure also begins with the administration of the cytostatic drug, but the supply of electric pulses starts after 8 min [20,43]. When considering the half-life of bleomycin, the optimal concentration of the drug is maintained in the bloodstream between 8 and 28 min after administration. The updated ESOPE extended this gap to 45 min [20,43].

## 4. Advantages, Contraindications, Side Effects and Limitations of ECT

The procedure is not expensive and is easily performed. A suitable cabinet for preparation and treatment is enough if the procedure is created by local anesthesia. Patients do not require hospitalization after the treatment; they only wait for a few hours in the department if the need for special medical attendance arises. Most ECT treated patients answered in a clinical trial that they would accept the procedure again if necessary [20,43]. Since the effect is mainly tumoral and the chemotherapeutic drugs reach low systemic concentrations, the incidence of systemic side effects is significantly reduced. Therefore, the treatment is suitable for elderly people and patients in poor physical condition, even with repeated ECT sessions [43,48]; however, caution should be maintained since neurological complications have rarely been reported [49].

There are only a few contraindications for performing ECT, but they need to be considered before recommending the procedure to an already suffering patient. ECT cannot be recommended to patients with renal failure, interstitial lung fibrosis (when using BLM), epilepsy, a pacemaker, or an allergy to the administered drug [50,51].

It has low toxicity and minor complications. The main side effects are local and transient, including local pain, swelling, redness, depigmentation or hyperpigmentation, muscle contractions during electroporation [52], and ulcers (when an exophytic tumor necroses) [26,53]. While there are scarce reports in the literature on the neurological and/or cerebrovascular complications of ECT, Landstrom et al. (2021) recently raised awareness by reporting severe events in patients with head and neck ECT, possibly associated with the procedure (one seizure and one fatal ischemic stroke) [49].

Although the procedure barely has any restrictions, some limitations still occur. The important point is that no arrhythmias or other pathological changes in the ECG recordings during ECT have been found [54]. Specific treatment protocols are available for individual tissues to improve the quality of the result. The type of electrode needs to be chosen according to the type of tumor and individual pulse generators [55]. Tumors larger than 3 cm have a lower response to ECT compared with nodules smaller than 1 cm^2^. When a tumor responds only partially, it can be retreated after 4 weeks or anytime needed with no loss of ECT efficacy [56]. If the treated area was irradiated or contained fibrotic tissue, the penetration of the electrode might be impaired; consequently, a suboptimal amount of drug or electrical current will be delivered [57]. When the number of tumors is limited, and their size does not exceed 3 cm in diameter, the result is optimal, but it can also be efficient in patients with up to 15 skin metastases [50]. When more nodules are present, there is a need for more treatment sessions. As expected, patients treated with ECT show fewer side effects than patients treated with systemic chemotherapy. ECT can also be used as adjuvant therapy [58].

## 5. Clinical Applications of ECT in Melanoma

Electrochemotherapy is used not only for cutaneous metastasis but also for primary tumors [59]. Patients who underwent cardiac surgery, radiofrequency ablation (RFA), transarterial chemoembolization (TACE), or who have comorbidities such as diabetes can be treated by ECT [60,61]. Its efficacy is well demonstrated for cutaneous and subcutaneous primary and metastatic melanomas [62], primary and metastatic basal cell carcinoma [63,64], primary and metastatic squamous cell carcinoma [65,66], keratoacanthoma [67], ungual warts [68], Kaposi’s sarcoma [3,69], Merkel cell carcinoma [70], cutaneous primary and metastatic lesions of breast cancer [62,71], soft tissue sarcoma (STS) [72], cutaneous B-cell lymphoma [73,74], superficial angiosarcoma [75], locally advanced and metastatic angiosarcoma [76], and as palliative therapy for tumor complications [77,78,79].

ECT made its clinical debut in treating melanoma tumors [80,81] but expanded quickly to various histological cutaneous tumors. Our paper summarizes multiple clinical trials on ECT and electroporation of targeted therapies in melanoma (Table 1).

In a clinical trial (NCT00006035), DeConti et al. (2000) aimed to study the effectiveness of ECT with intratumoral bleomycin in stage III or IV melanoma patients [82]. Other objectives were to determine the safety of electroporation therapy, to compare the healing time and the duration of lesion response with these treatments in the selected group of patients [82]. The results have not yet been published.

Ricotti et al. (2014) proposed the role of ECT as a first-line palliative treatment in metastatic melanoma [83]. The group conducted a clinical study that recruited 30 patients with 654 cutaneous and subcutaneous melanoma metastatic nodules who were treated with intravenous bleomycin ECT [83]; the results were: 100% objective response rate (67.28% with complete response and 32.72% with partial response) and 72% local tumor control rate after 24 months [83].

Kunte et al. (2017) conducted a prospective cohort study on 151 patients with metastatic melanoma identified from the International Network for Sharing Practices on Electrochemotherapy) database [84]. The treatment was well-tolerated with a complete response in 58% of lesions (229/394). This result is significantly associated with several factors: tumors less than 3 cm, coverage of deep margins, and a history of irradiation of the treated area [84].

At the beginning of 2018, Ferrucci et al. initiated a phase II multicenter, open-label, non-randomized, interventional study (NCT03448666), with an estimated enrollment of 53 patients suffering from unresectable melanoma with superficial or superficial and visceral metastases [85]. The hypothesis of the clinical study is that concomitant pembrolizumab and ECT treatments are safe and capable of improving local and systemic response rates [85]. ECT will be performed with the cliniporator and a single intravenous dose of bleomycin [85]. The first results are expected for 2023 [85].

Kis et al. conducted a randomized phase II clinical trial (NCT03628417), initiated in 2018 [86] with published results in 2020 [87], that compared the effect of calcium electroporation with bleomycin-based ECT on cutaneous metastases of any histology, including melanoma [86,87]. An important result showed that calcium electroporation and ECT were associated with a release of High Mobility Group Box 1 protein (HMGB1) in vitro (*p* = 0.029) and a significant increase in the overall systemic level of proinflammatory cytokines in serum from the treated mice (*p* < 0.003) [86,87]. These findings indicate that calcium electroporation, as well as ECT, may have a role as immune stimulators in future treatments [86,87].

In a randomized double-blinded phase II study, Falk et al. (2018) also showed good results for both bleomycin-based ECT (84%; 16/19) and calcium electroporation (72%; 13/18), with superiority of the first [59].

In a single-arm phase-2 study (ISRCTN.11667954), Simioni et al. (2020) offered an alternative to the standard procedure of ECT that has the disadvantage of being limited to the treatment of superficial tumors: the variable electrode-geometry ECT [88]. It uses an innovative, longer, freely-placeable electrode for soft-tissue deep-seated malignancies, including melanoma [88].

**Table 1:** Clinical applications of electrochemotherapy (ECT) in melanoma.

No	Author	Clinical Trial Number	Therapeutic Agent	Reference
1	DeConti et al.	NCT00006035	Intratumoral bleomycin	[82]
2	Ricotti et al.	-	Intravenous bleomycin	[83]
3	Kunte et al.	-	Intratumoral or intravenous bleomycin	[84]
4	Ferrucci et al.	NCT03448666	Pembrolizumab and intravenous bleomycin ECT	[85]
5	Kis et al.	NCT03628417	Intratumoral calcium electroporation vs. intratumoral bleomycin ECT	[87,88]
6	Falk et al.		Intratumoral calcium electroporation vs. intratumoral bleomycin ECT	[59]
7	Simioni et al.	ISRCTN.11667954	Intravenous bleomycin with variable electrode-geometry ECT (VEG-ECT)	[88]

Source: Summarized Section 5 from text.

## 6. Other Clinical Applications of Electroporation in Melanoma

The good results in the management of metastatic melanoma of ECT and the need for therapeutic alternatives have extended the area of research toward the delivery by electroporation of targeted therapies such as gene transfer or immunotherapy as can be seen in Table 2.

### 6.1. Intralesional Gene Transfer by Electroporation of Interleukin Plasmids

Interleukin 12 (IL-12) is an important regulatory molecule of the innate and adaptive immune responses, with proven clinical use in the treatment of solid malignancies, including melanoma [89,90]. The systemic administration of IL-12 was associated with severe adverse reactions and potentially life-threatening toxicity [89,90]. On the other hand, the intratumoral delivery of IL12 through plasmid electroporation showed significantly lower toxicity [89,90]. Several clinical trials studied the efficacy and safety of intratumoral delivery of plasmid encoding IL12 through electroporation in melanoma.

Daud et al. (2008) were among the first to conduct a human trial of gene transfer utilizing in vivo DNA electroporation [91,92]. The group aimed to establish the maximum tolerated dose of intralesional electroporated IL-12 plasmid for patients with metastatic melanoma [91,92]. In the study (NCT00323206), patients received plasmid IL-12 intra-tumoral, followed by electroporation, leading to the accumulation of plasmid DNA in the malignant cells. The results were promising since two patients of 19 with nonelectroporated distant lesions and no other systemic therapy showed complete regression of all metastases, and the other eight showed disease stabilization or partial response [91,92].

Algazi et al. (2011) completed an open-label phase II clinical trial for advanced melanoma patients (NCT01502293) [93]. In order to induce an inflammatory response within the tumors and further initiate and/or enhance anti-tumor immunity, the patients were treated with plasmid encoding IL-12 (tavokinogene telseplasmid-tavo) followed by electroporation [93]. Although the treatment was well-tolerated by the patients, the response was limited due to adaptive immune resistance [93,94,95].

Tsay K et al. initiated a multicenter, phase II, open-label, (NCT02493361) study in 2015 that evaluated 42 patients with melanoma treated with intratumoral pIL-12 electroporation in combination with pembrolizumab [96]. The patients’ responses were strictly evaluated in two parts. Pembrolizumab was provided intravenously on the first day of each cycle and pIL-12 was injected into the tumor [96]. This treatment combination with electroporation led to a significant reduction in the lesion dimension [96].

Another extensive phase II trial (NCT03132675) led by Malloy et al. (2017) analyzed the efficiency of intratumoral tavokinogene telseplasmid (tavo; pIL-12) electroporation plus intravenous pembrolizumab [97]. The eligibility criteria included patients diagnosed with unresectable or metastatic melanoma that is progressing or have progressed on pembrolizumab or nivolumab [97]. The results have not yet been published.

In 2008 Kharkevitch et al. included patients diagnosed with metastatic melanoma in the clinical trial (NCT00223899), studying the safety and effects of intratumorally injected VCL-IM01 followed by electroporation [98]. VCL-IM01 is an IL-2-encoding plasmid. Response rate, duration of response and cutaneous adverse reactions were assessed [98]. The results have not yet been published.

### 6.2. Gene Transfer of Human Telomerase Reverse Transcriptase (hTERT) DNA Plasmid

A phase I clinical trial (2008) (NCT00753415) investigated the safety, tolerability, and immune response for V934-EP/V935 vaccine in patients with multiple solid tumors, including stage IIB or III melanoma [99]. The other trial investigated solid tumors that were non-small-cell lung carcinoma, breast cancer, upper gastrointestinal tract carcinoma, colon carcinoma, renal cell carcinoma, bladder carcinoma, and prostate cancer [99]. V935 is an adenoviral type 6 vector vaccine expressing a modified version of human telomerase reverse transcriptase (hTERT), while V934 is an hTERT DNA plasmid delivered using the electroporation injection technique. The two vaccines were administered alone or in combination, either in a low dose or high dose [99,100]. The results suggested the safety and feasibility of V934-EP/V935 hTERT vaccination in melanoma patients [99,100].

### 6.3. Gene Transfer of Tyrosinase DNA Plasmid

Wolchok et al. (2010) aimed to determine the safety and feasibility of electroporation mediated intramuscular delivery of tyrosinase DNA plasmid vaccine in patients with melanoma (NCT00471133) [101]. The magnitude and frequency of tyrosinase-specific immunologic responses in the immunized patients were also assessed [101]. The conclusion showed that a regimen of five immunizations administered by electroporation is safe and provides an efficient immune response [101,102].

### 6.4. SCIB 1—A Human Immunoglobulin G1 Antibody DNA Vaccine

Lorigan et al. (2010) aimed to investigate a novel immunotherapy, SCIB1, for the treatment of melanoma (NCT01138410) [103]. SCIB1 is a melanoma DNA vaccine that incorporates specific epitopes from the proteins gp100 and TRP-2 within an antibody framework (ImmunoBody^®^), aiming to stimulate the patient’s T cells to emit a specific response to melanoma cells [103,104,105]. The aqueous solution of plasmid DNA was administered intramuscular using the TDS-IM electroporation device [103]. The safety, tolerability, and immunological effects of SCIB1, as well as the performance of the injection device were evaluated [103]. The results have not yet been published.

Patel et al. (2019) are currently recruiting patients for an interventional, open-label, uncontrolled study (NCT04079166) with the purpose of finding if a new treatment called SCIB1 can be used safely when added to Pembrolizumab [106]. The study will also try to determine if SCIB1 increases the response to pembrolizumab and if SCIB1 improves the length of response [106].

### 6.5. Gene Transfer of Antiangiogenic Metargidin Peptide Plasmid

AMEP (antiangiogenic metargidin peptide) is an antiproliferative and antiangiogenic molecule that binds to *αvβ3* and *α5β1* integrins [107].

Vasseur et al. started a study in 2012 (NCT01764009) that aimed to determine the dose limiting toxicity (DLT), maximal tolerated dose (MTD), efficacy, local and general safety of intramuscular electrotransferred plasmid AMEP in patients with advanced or metastatic melanoma [108]. Unfortunately, the study was withdrawn in 2015 due to the low enrollment rate.

Pierre et al. conducted a study (NCT01045915) that aimed to evaluate the local and general safety of the intratumoral electrotransfer of escalating doses of plasmid AMEP in patients suffering from advanced or metastatic melanoma [109]. The team also intended to identify specific doses that may be effective on cutaneous lesions [109]. In this study, the increasing doses were administered at one-week intervals [109]. The study has been halted due to the low enrollment rate.

Spanggaard et al. (2013) conducted this first-in-man phase I study on five patients with disseminated melanoma, who had no further treatment options, treated with the electroporation of AMEP plasmid [107,110]. While related serious adverse events did not occur (only transient fever and elevated C reactive protein levels), after 29 days, there was no reduction of tumor size, but rather stability or progression [107].

### 6.6. mRNA Electroporated Autologous Dendritic Cells

Young et al. (2011) from Memorial Sloan Kettering Cancer Center initiated a single-arm phase I trial (NCT01456104) concerning the immune responses to autologous Langerhans-type dendritic cells electroporated with mRNA encoding a tumor-associated antigen in patients with melanoma [111]. The purpose of this study was to determine if the immune system can fight against melanoma [111]. In a physiological background, dendritic cells lack cancer proteins on their surface. Theoretically, combining the antigens with dendritic cells will produce a vaccine capable of activating the body’s T cells, resulting in tumoral destruction [111]. The mRNA is introduced into the dendritic cells by electroporation, thereby showing the malignant antigen on their surface [111]. Using this method, the body develops a stronger immune response against melanoma [111,112].

In another clinical trial (NCT01676779), Neyns et al. (2012) conducted a phase II randomized controlled trial in patients with melanoma stage IIIB/C and IV [113]. The patients were divided into two arms: The first arm received mRNA electroporated autologous dendritic cells therapy for one year, the second arm initiated dendritic cell therapy only after the recurrence of the melanoma that could not be managed with local therapy [113]. The results have not yet been published.

Punt et al. (2014) completed a study (NCT01530698) investigating the immunological response upon vaccination with TLR-dendritic cells versus Trimix dendritic cells, loaded with mRNA encoding melanoma-associated tumor antigens [114]. The study assessed the efficacy of different doses of vaccines and related toxicity [114]. The results have not yet been published.

**Table 2:** Other clinical applications of electroporation in melanoma. IL—interleukin; hTERT—human telomerase reverse transcriptase; AMEP—antiangiogenic metargidin peptide plasmid.

No	Author	Clinical Trial Number	Therapeutic Agent	Reference
Intralesional gene transfer by electroporation of interleukin plasmids				
1	Daud et al.	NCT00323206	IL-12p DNA	[91,92]
2	Algazi et al.	NCT01502293	Tavokinogene telseplasmid (IL-12p)	[93,94,95]
3	Tsay et al.	NCT02493361	IL-12p and pembrolizumab	[96]
4	Malloy et al.	NCT03132675	Tavokinogene telseplasmid (IL-12p) and pembrolizumab	[97]
5	Kharkevitch et al.	NCT00223899	VCL-IM01 (IL-2 encoding plasmid)	[98]
Gene transfer of human telomerase reverse transcriptase (hTERT) DNA plasmid				
6	Aurisicchio et al.	NCT00753415	V934-EP/V935 vaccine	[99,100]
Gene transfer of tyrosinase DNA plasmid				
7	Wolchok et al.	NCT00471133	Intramuscular tyrosinase DNA plasmid vaccine	[101]
SCIB 1—a human immunoglobulin G1 antibody DNA vaccine				
8	Lorigan et al.	NCT01138410	Intramuscular SCIB1	[103,104,105]
9	Patel et al.	NCT04079166	Intramuscular SCIB1 and pembrolizumab	[106]
Gene transfer of antiangiogenic metargidin peptide plasmid				
10	Vasseur et al.	NCT01764009	Intramuscular AMEP	[108]
11	Pierre et al.	NCT01045915	Intratumoral AMEP	[109]
12	Spanggaard et al.	-	Intratumoral AMEP	[107,110]
mRNA electroporated autologous dendritic cells				
13	Young et al.	NCT01456104	Subcutaneous autologous Langerhans-type dendritic cells electroporated with mRNA encoding a melanoma-associated antigen	[111,112]
14	Neyns et al.	NCT01676779	Intravenous and intradermal mRNA electroporated autologous dendritic cells	[113]
15	Punt et al.	NCT01530698	Intranodal trimix dendritic cells electroporated with mRNA encoding melanoma-associated antigens	[114]

Source: Summarized Section 6 from text.

## 7. Conclusions

ECT is a local efficient procedure that improves the quality of life and morbidity for patients through tissue-sparing. Due to the low dose of chemotherapy, ECT does not imply high risks, has the advantage of the absence of surgical wounds, good aesthetic results, reduced hospitalization time, and high patient compliance. ECT has only a few adverse effects and is efficient in more than half of patients with advanced tumors. In the case of melanoma, ECT is recommended both for curative and palliative purposes, with promising response rates.

The intratumoral delivery of targeted therapies by electroporation represents a viable alternative to the systemic administration associated with increased toxicity. Intratumoral gene transfer through electroporation offers valuable therapeutic options in advanced melanoma, either alone or in combination with systemic agents.

An interdisciplinary approach is recommended when using ECT. The collaboration of dermatologists, surgeons, oncologists, and eventually anesthesiologists is compulsory because current treatment modalities are based on a multimodal perspective.

## Data Availability

Not applicable.
